# Combined action observation and motor imagery therapy: a novel method for post-stroke motor rehabilitation

**DOI:** 10.3934/Neuroscience.2018.4.236

**Published:** 2018-12-21

**Authors:** Jonathan R. Emerson, Jack A. Binks, Matthew W. Scott, Ryan P. W. Kenny, Daniel L. Eaves

**Affiliations:** School of Health and Social Care, Teesside University, Middlesbrough, UK

**Keywords:** neurorehabilitation, mental practice, combined action observation and motor imagery, AO + MI, motor simulation, demonstrations, motor recovery, action representation, stroke, motor impairment

## Abstract

Cerebral vascular accidents (strokes) are a leading cause of motor deficiency in millions of people worldwide. While a complex range of biological systems is affected following a stroke, in this paper we focus primarily on impairments of the motor system and the recovery of motor skills. We briefly review research that has assessed two types of mental practice, which are currently recommended in stroke rehabilitation. Namely, action observation (AO) therapy and motor imagery (MI) training. We highlight the strengths and limitations in both techniques, before making the case for combined action observation and motor imagery (AO + MI) therapy as a potentially more effective method. This is based on a growing body of multimodal brain imaging research showing advantages for combined AO + MI instructions over the two separate methods of AO and MI. Finally, we offer a series of suggestions and considerations for how combined AO + MI therapy could be employed in neurorehabilitation.

## 1. Introduction

Cerebral vascular accidents (strokes) are a leading cause of motor deficiency in millions of people worldwide [1]. Unfortunately, the number of people affected by stroke will inevitably rise as global life expectancy increases. The prevalence of motor deficits following a stroke can be up to 80% in a defined elderly population [2]–[3]. Only a small proportion of this group (approximately 20%) will partially recover from impaired motor ability, leaving approximately 50–60% who are left with some form of chronic motor deficiency [3]–[4]. Motor impairments are defined as the loss or restriction of function in the control of muscles, movements, or mobility [5]. Areas frequently affected are the hand, arm, face and leg, typically on one side of the body [1]. Motor impairments may involve disordered sensorimotor and proprioceptive control, which often result from an upper motor neuron lesion, as evidenced by intermittent or sustained involuntary muscle activations [6]. Hemiparesis or muscle weakness [7] is also commonly observed, resulting in altered movement patterns in both the contralateral and ipsilateral side (with respect to the side of the lesion, paretic or nonparetic [8]).

Encouragingly, there is strong evidence in cognitive neuroscience literature showing the brain can adapt or reorganise itself in response to sensory input, learning and experience [9]. This process is referred to as *neuroplasticity* [10], and can occur in both the healthy and impaired brain [11]–[12]. Following a stroke, neurorehabilitation aims to reduce motor deficits via neuroplasticity effects, typically through repetitive physical training, constraint-induced movement therapy, or physical therapy [13]–[15]. Several useful approaches highlighted in the literature also include: mirror box therapy, electrical stimulation (e.g., non-invasive brain stimulation or vagus nerve stimulation), fitness training, biofeedback and robotic interactions [16]. Given that everyday movements can be significantly impaired, particularly in the acute post-stroke phases, physical practice may not be possible or appropriate. To address this pertinent issue, a great deal of research has investigated the efficacy of mental practice techniques for enhancing neurorehabilitation instead [17].

In this paper our aim is not to provide a review of literature that is either exhaustive or fully systematic in nature. Instead, we provide a balanced and concise analysis and discussion of contemporary research examining the use of two mental practice techniques, namely, action observation (AO) and motor imagery (MI) in post-stroke rehabilitation. First, we briefly review some of the key studies investigating the benefits and limitations of using both AO and MI independently in stroke rehabilitation. We refer to the evidence supporting neuroplasticity effects, which are argued to underpin the rehabilitation process. We also address the practical and applied implications for the delivery of both AO and MI in clinical and home settings. Where possible we also make reference to published guidelines and recommendations for stroke rehabilitation.

We then review all available research to date that has investigated the effects of combined action observation and motor imagery (AO + MI) instructions, predominantly in healthy adults. This section covers a growing body of multimodal brain imaging research, and a collection of carefully designed behavioural studies. We give particular focus to the one study that has investigated combined AO + MI therapy in stroke rehabilitation [18]. While the main thrust of our argument is in favour of the advantages in this approach, we highlight the potential limitations of using combined AO + MI therapy with regards to a patient's cognitive ability and lesion location. To address some of these limitations, we suggest a method of carefully layering AO and MI practices over time, in line with the progressive nature of the rehabilitation process, to build toward a more complex and integrated form of AO + MI therapy, which could later be combined with physical rehabilitation.

Finally, we offer a series of suggestions and considerations for how combined AO + MI therapy could be incorporated into existing neurorehabilitation practice. The aim of this section is simply to provide a series of examples and scenarios to stimulate debate and discussion in the field about the suitability of AO + MI therapy in different contexts.

## 2. Neurorehabilitation via different forms of motor simulation

Action observation (AO) therapy and motor imagery (MI) training are two prominent mental practice techniques that have both been recommended as potentially effective intervention tools [19]. AO refers to the deliberate and structured observation of human movement [20], whereas MI is defined as “the mental representation of action without any [overt] concomitant body movement” [21]. The rationale for using these two methods is predicated on multimodal neuroimaging evidence, showing the brain areas involved in AO at least partially overlap with those involved during MI. Moreover, the brain regions activated during both AO and MI overlap extensively with those involved in motor execution [22]. On these grounds, Jeannerod's [23] influential hypothesis was that AO and MI can be regarded as two forms of motor simulation that are functionally equivalent to each other. Despite this early integrative account, it is surprising that both researchers and practitioners have largely employed either AO or MI methods in isolation from each other [24].

### 2.1. Action observation therapy

The efficacy of action observation (AO) therapy in neurorehabilitation is well grounded in neurophysiological research. Substantial evidence has confirmed that seeing an action can generate an internal representation of the observed action [25], which can prime execution of the same action [26]. The neuronal substrate for this has been termed the mirror neuron system [25]. On this basis, AO therapy involves the systematic observation of actions prior to execution [27]. For example, reaching, grasping, transporting and perhaps manipulating an object, before finally releasing the grip. Following each observed action, patients physically execute the same action and sessions are repeated daily. Research shows AO therapy can yield significant improvements in upper limb movement in ischaemic stroke patients, over a four week period, relative to both the baseline and a control group [28]–[29]. These improvements are typically retained for several months post intervention.

In particular, Ertelt et al.'s [28] functional magnetic resonance imaging (fMRI) study reported a significant rise in the neurophysiological activity recorded in premotor regions of the cortex. This increase was specifically in those stroke patients who were allocated to the AO therapy group, compared to those in the control groups. The brain regions exhibiting neuroplasticity effects included: the bilateral ventral premotor cortex, bilateral superior temporal gyrus, the supplementary motor area, and the contralateral supramarginal gyrus. Accordingly, those authors argued that the mirror neuron system's capacity to re-enact observed actions should be harnessed to promote rehabilitation in motor regions of the brain, and facilitate neuroplasticity effects [30]. Buccino [27] recently argued that future research should therefore consider how to combine AO therapy effectively with other methods in neurorehabilitation.

Live demonstrations are easily administered by practitioners in face-to-face clinical settings, wherein AO therapy would not normally not involve any particular skill on the patient's side. This assumption is based on substantial neurophysiological evidence showing that even passive action observation can activate an associated motor representation in the observer's brain [25]. To create home-based AO therapy would instead require a library of action videos. It would also be necessary for the patient to have both the access and capacity to engage with the technology that displays these videos within their home. Regardless of the location for delivery, however, a limitation in the current delivery method for AO therapy is that practitioners will rarely instruct a patient on either *how* they should observe the target action, or *what* features they should focus on. This is in contrast with MI training, which involves specific instructions for action content. For MI the vividness of the experience depends on each patient's ability to form and maintain the required motor simulation [31]–[32]. Moreover, while Gatti et al. [19] showed increased motor involvement for AO compared to MI, a number of studies have shown the reverse trend [24].

### 2.2. Motor imagery training

Motor imagery involves simulating the visual and kinaesthetic aspects of an action without any overt movement occurring [33]. This can be undertaken either from an internal, first person, perspective (as if performing the imagined movement from one's own point of view) or an external, third person, perspective (as if watching oneself making the movement form outside of the body). Furthermore, internal kinaesthetic imagery should be dissociated from internal visual imagery. This is on the basis that the former modulates corticospinal excitability at the supraspinal level, while the latter does not [34]. The kinaesthetic and first person internal perspective are also best measured using separate subscales in self-report questionnaires, reflecting the academic consensus for a more differentiated approach to assessing MI ability [35].

A large body of research shows MI training has the potential to improve motor abilities in neurorehabilitation [36], and that the neural reorganisation following MI training is similar to the changes following physical training [37]–[38]. In a landmark fMRI study into the neuroplasticity effects associated with MI training in stroke patients, Sharma et al. [39] reported three key findings. First, after subcortical stroke there was evidence for cortical neuroplasticity, both in terms of regional activation, and connectivity within distributed brain networks. These changes in network connectivity were observed most clearly during MI. Second, plasticity occurred in cortical areas that were remote from the subcortical infarct. Cortical changes were therefore not the result of local cortical injury, but a response to injury elsewhere in the motor system. Third, the changes in connectivity during MI correlated with motor function. A more recent study has also shown acute MI training not only modulates plasticity effects at the cortical level, but also further downstream at the level of spinal presynaptic inhibition, reflecting the sensitivity of the spinal circuitry to MI [40].

A systematic review by Zimmermann-Schlatter et al. [41] initially reported there was moderate evidence supporting the use of MI in conventional physiotherapy for stroke rehabilitation. They concluded MI is something easily learned, requires no physical effort and poses no harm to the patient. A more recent meta-analysis by Machado et al. [42] focused on randomized controlled trials that assessed imagery training effects. Those authors instead argued the evidence for imagery benefits in stroke rehabilitation is at best only mixed [36]. Despite contradictions like this in the evidence base, published guidelines from The American Heart Association, American Stroke Association [43] and the Canadian Stroke Best Practice recommendations [44] all state MI should be included alongside physical practice to maximise stroke rehabilitation. Bovend'Eerdt and colleagues [45] have also provided an evidence-based guide for including MI in neurorehabilitation in a clinical setting. While these guides are constructive and informative, there are many areas of MI use in stroke rehabilitation that require further investigation.

For example, it is not yet clear what the optimal dosage for MI should be over the duration of a rehabilitation programme, and how this should be tailored to the individual. Previous efforts to define clinically optimal MI dosage effects on motor recovery include assessments for both twenty and sixty minute MI sessions, delivered three days a week for ten weeks [46]. Completing twenty minute sessions led to an increase in scores for the Action Research Arm Test (ARAT), while sixty minute sessions led to an increase in Fugl-Meyer Assessment (FMA) scores. Although these findings suggest MI can improve motor function, the assessment method should also be considered in terms of how the desired outcome is defined.

Another issue is that stroke patients can have difficulties in generating MI from verbal instructions in a clinical setting. In particular, cognitive impairments caused by a stroke can make verbal instructions and cues difficult to follow [47]. While it is not currently clear how existing approaches to MI training can avoid this problem, combined AO + MI therapy may offer potential solutions (discussed below). Future work must therefore further investigate the imagery training methods, delivery, format and scheduling best suited within stroke rehabilitation.

An intuitive advantage of MI is that this method is cost-free and can easily be incorporated throughout all stages of stroke recovery and existing physical rehabilitation programmes. Patients may also be able to practice MI independently between their physical therapy sessions. Furthermore, stroke patients can be encouraged to imagine performing actions or tasks that they are currently unable to physically achieve due to their impaired motor abilities.

Physical rehabilitation primarily focuses on the recovery of previously learned actions (i.e., motor re-learning) instead of training novel movements. It is crucial practitioners take the same approach in MI training. During physical practice, brain activity in healthy adults is typically more bilateral and diffuse across hemispheres when the task is novel compared to well-practiced [48]. In MI, this pattern of cortical activity is similarly more diffuse for imagery of un-practiced actions compared to imagery of familiar actions and, overall, cortical activity is more diffuse and bilateral for imagery compared to execution [49]. Accordingly, it can be difficult to learn novel actions via MI alone, as the task-related neural networks may be absent, unorganised [50], or damaged in the case of stroke patients. Research shows that cortical activity reduces in task specific brain regions as a function of physical practice and this effect can also occur, though to a lesser extent, using imagery [51]. Imagery based rehabilitation therapy must therefore incorporate actions that will encourage both the growth and refinement of these neural networks to support physical gains. Practitioners should also avoid making the MI task too challenging for the patient, considering both the severity of the stroke and the patient's motor repertoire prior to the stroke.

## 3. Combined action observation and motor imagery therapy

Traditionally, the two forms of motor simulation (that is, AO and MI) have been studied either in isolation from each other, or compared, in terms of their relative effectiveness in rehabilitation [19],[24],[47]. More recently, however, a growing number of studies has investigated the potential advantages of instructing combined action observation and motor imagery (AO + MI; [24],[33]). This instruction involves imagining the kinaesthetic experience and sensations of action, while also observing a visual display of the same action at the same time [33]. Participants are normally instructed to synchronise their imagined action with the observed movement in real time. Despite the obvious need to verbally describe this task in the first instance, showing patients a visual representation of the target action could help to reduce the volume of instructions needed to cue imagery of the same action. This would therefore negate some of the problems inherent in stroke patient treatment, regarding their ability to understand and follow the verbal cues associated with traditional imagery training [47].

Multimodal brain imaging research now provides remarkably positive and consistent evidence showing combined AO + MI instructions can significantly increase the level of neurophysiological activation in cortico-motor regions of the brain, compared to either purely imagining or observing the same action [33]. This effect has been reliably demonstrated across the following neurophysiological measures: fMRI [52]–[55], electroencephalography (EEG; [56]–[59]) and transcranial magnetic stimulation (TMS; [60]–[65]). Given the main premise for undertaking both AO therapy and MI training is the increase in motor involvement compared to baseline and control conditions, it is striking that this facilitation in motor activity is significantly more pronounced, and in some cases in a super-additive way [55], for combined AO + MI instructions. Accordingly, this pattern of cortical activity in motor and motor-related regions of the brain more closely represents the motor system's involvement during physical execution [52]. In turn this would suggest clear advantages for combined AO + MI instructions in rehabilitation over separate AO and MI methods.

While those neurophysiological studies focused on healthy adult participants, the authors have regularly recommended combined AO + MI training for neurorehabilitation purposes. This is an intuitive suggestion that may hold promise for a range of brain injured patients, including those recovering from a stroke. The related behavioural research in a rehabilitation setting, however, is currently sparse. So far, a series of behavioural studies has shown significant advantages for combined AO + MI instructions in healthy adults. This was during balance training [66], instantaneous and automatic imitation [58],[67]–[68], peak force development [69], target aiming [70], and accelerated rehabilitation post hip arthroplasty [71]. A recent study has also shown significantly greater modulations in movement kinematics for combined AO + MI instructions in Parkinson's disease patients, compared to AO instructions [72]. An open question is whether gains in similar tasks will also be possible in stroke patients. To our knowledge, Sun et al. [18] was the first, and remains the only experiment to investigate a combined AO + MI therapy intervention in stroke patients.

The study by Sun and colleagues [18] included ten right handed participants who all had a stroke lesion affecting their left hemisphere, resulting in impaired right arm function. All participants received the same conventional physical rehabilitation on a daily basis for four weeks, while undertaking five rounds of mental practice during this period. Patients were asked to imagine the effort and sensations of moving their right hand to grasp, lift, and insert a small peg into a hole on a board, before removing the peg from the hole. Half of the participants imagined this while they simultaneously observed the same action displayed on a screen (i.e., combined AO + MI therapy). In this condition, the observed action was intended as a visual guide to help refine and maintain the concurrently imagined action. The other half (control group) observed and then subsequently imagined the same action, that is, in a serial fashion, rather than in a combined way. For this group, the visual guide was intended as a method of priming the subsequently imagined action.

Sun et al. [18] reported significant improvements in two measures of motor ability: the FMA and pinch grip strength test. A clinically important improvement in FMA scores is defined as between 4.25 to 7.25 points [46]. In Sun et al.'s [18] study, both the combined AO + MI and the control group improved their FMA scores by a clinically important margin over the four week period (24.8 and 19.6, respectively). Indeed, the difference between the two groups at the end of the testing period could also be considered clinically meaningful, demonstrating combined AO + MI therapy can improve upper extremity motor function in post-stroke patients (see Table 1).

**Table 1.** Data adapted from the study published by Sun et al. [18]. Mean scores for the Fugl-Meyer Assessment, reported over a four week period for two post-stroke rehabilitation groups: action observation followed by motor imagery vs. combined action observation and motor imagery.

	Baseline	Week 1	Week 2	Week 3	Post-test	Change score
Action observation followed by motor imagery	14.8	19	24	29.6	34.4	19.6
Combined action observation and motor imagery	14.6	20.6	27.8	33	39.4	24.8
Between-group difference	0.2	1.6	3.8	3.4	5	5.2

Sun et al. [18] also reported EEG recordings over the primary sensorimotor cortex. Event-related desynchronization of the mu rhythm was greater in amplitude and duration for the combined AO + MI group, reflecting enhanced cortico-motor involvement in response to this training method. Although this study was both limited to a relatively small sample size, and did not include a strict control group, it does provide the first evidence indicating combined AO + MI therapy can facilitate neuroplasticity effects to support motor recovery within stroke patients more effectively than AO followed by MI.

One possibility, which now requires further investigation (for e.g., using structural modelling techniques), is that these changes for combined AO + MI therapy reflect a modulation of intracortical and subcortical excitatory mechanisms, through synaptic and cortical plasticity. In this context, the changes reported in Sun et al.'s [18] study are likely to be driven by mechanisms that more closely resemble those involved during physical practice of the same task, which in turn would be expected to produce gains that are greater in magnitude over and above those obtained using either AO or MI [47].

Overall, this growing body of multimodal neuroimaging evidence indicates that it is possible for healthy adults to co-represent both an observed and an imagined action simultaneously (termed ‘dual-action’ simulation [33],[58]). Presumably, this is in the form of two parallel sensorimotor streams. A useful framework for conceptualising such dual-action simulation can be formulated on the basis of Cisek and Kalaska's [73] affordance competition hypothesis. Their model describes how multiple sensorimotor representations are maintained in parallel, as a set of action affordances. Preparation for action would then involve different brain areas submitting their ‘votes’ to contribute toward the selection of available movement parameters, prior to movement execution. In the context of combined AO + MI therapy, concurrent representations involving an observed and an imagined action would be maintained simultaneously, and these two streams would either merge or compete, based on their content and relevance towards ongoing action plans [33],[58],[68],[74]. While the efficacy of this dual-action simulation hypothesis is yet to be comprehensively explored both in the healthy brain, and the related motor behaviour, the application of this conceptual lens will likely offer novel approaches to rehabilitation of the injured brain.

### 3.1. Potential limitations of combined AO + MI therapy

An important point when considering combined AO + MI therapy in stroke patients is that some (though not all) of the limitations that currently apply to the use of MI could also apply to combined AO + MI therapy. The primary issue concerns whether a stroke patient is indeed capable of engaging in imagery at all. Several studies have shown mental imagery is multifaceted, involving a number of different cognitive functions and brain areas [31]. Visual compared to motor imagery for instance involves activations in different neuronal subsystems. There is also sufficient evidence for individual differences underlying imagery ability, based on differences in brain structure [35].

As mentioned earlier, there is currently mixed support for the efficacy of MI training in stroke rehabilitation. One explanation for the discrepancies in the literature may be the lack of control for MI ability. The key issue is that the particular location of damage in a patient's brain may prevent successful MI performance, and this may account for the absence of an MI-based treatment effect [75]. Research has established that impairments in parietal lobe function, either through stroke [76] or inhibitory brain stimulation in healthy participants [77]–[78] significantly reduces MI ability. Moreover, Oostra et al.'s [76] study highlighted the importance of an intact fronto-parietal network for MI, and the crucial role of the basal ganglia and premotor cortex when performing MI tasks. This was on the basis of their finding that low scores on a self-report MI vividness scale were associated with lesions in the left putamen, left ventral premotor cortex and long association fibres linking parieto-occipital regions with the dorsolateral premotor and prefrontal areas. Poor temporal congruence was otherwise linked to lesions in the more rostrally located white matter of the superior corona radiate [76].

In support of these findings, McInnes et al.'s [75] recent review and meta-analysis found broad evidence showing patients with parietal lobe damage were most impaired on their ability to perform MI. Damage to specific brain structures such as the parietal lobe, frontal lobe and basal ganglia consistently showed impairment in MI ability. Accordingly, McInnes and colleagues concluded that MI-based neurorehabilitation may not be efficacious in all patient populations.

While this conclusion is well grounded in terms of the limits inherent in a patient's imagery ability, as determined by the nature of the damage experienced in specific brain regions, the patient's rehabilitation outcomes may also be limited by virtue of traditional imagery training methods themselves. Neuroplasticity, or rewiring of motor networks, is the intended aim for the rehabilitation process, which ultimately strives to improve movement recovery rates [75]. The question remains, therefore, to what extent can combined AO + MI therapy help restore lost motor function in damaged brain regions, compared to traditional MI training methods? An increasingly persuasive rationale is that rehabilitation practitioners should attempt to capitalise on the significant increase in motor involvement associated with using combined AO + MI therapy, for the purpose of facilitating neuroplasticity.

A further challenge in stroke rehabilitation relates to the associated cognitive impairment. It is not within the scope of this article to review the extant literature on this substantial topic, but consideration of this evidence does have a strong bearing on the potential effectiveness of any modality for rehabilitation. Approximately 50% of stroke survivors are left with some form of cognitive impairment [79]. The affected cognitive domains are typically: attention and attention span, concentration, memory and decision making [80]. An important consideration for the use of combined AO + MI therapy in stroke rehabilitation therefore is the possible cognitive abilities participants might need to engage in this protocol.

Eaves et al. [58] recently showed that when healthy adult participants observed and concurrently imagined performing the same action there was a specific increase in the electrophysiological activity recorded over the rostral prefrontal cortex. Combined AO + MI methods may therefore come at an additional challenge to the user, in terms of the cognitive demands associated with supervisory control [58]. For stroke survivors, it will therefore be crucial to initially assess whether they are capable of undertaking AO + MI, in light of any cognitive impairments they may be experiencing.The preliminary evidence from Sun et al. [18] is that the stroke patients in their study were at least capable of achieving motor proficiency gains through combined AO + MI therapy, despite these additional cognitive demands. If patients can follow AO + MI instructions, then a fruitful avenue for future research is to investigate whether this method facilitates neural reorganisation effects in motor and motor-related areas, as well as in prefrontal regions. This suggestion is based on Jackson et al.'s [38] work, which similarly found neural reorganisation in prefrontal regions that correlated with increased physical performance following imagery combined with physical practice.

In summary, there are converging lines of evidence indicating combined AO + MI instructions may be more beneficial than the traditional approach of using AO and MI independently. This evidence comes from multimodal imaging studies, which show increased motor involvement for combined AO + MI instructions relative to the two individual techniques; as well as behavioural studies showing improvements in physical execution. Careful consideration should be given, however, to the potential constraints inherent in post-stroke brain function (on an individual patient basis), because this could impact the effectiveness of combined AO + MI therapy, in much the same way as it can impact the effectiveness of other therapies. There is now a rich opportunity for future research to investigate the extent to which combined AO + MI therapy could perhaps mitigate against these constraints, compared to other therapies, in clinical populations. One approach could be to examine neuroplasticity effects over time in the structural and functional brain mechanisms underpinning combined AO + MI instructions.

### 3.2. Integrating combined action observation and motor imagery therapy with existing methods

In this final section, we offer a series of proposals and considerations for how combined AO + MI therapy could be employed in neurorehabilitation. Our suggestions are intended primarily as illustrations to help stimulate debate and discussion in the field about the suitability of AO + MI therapy in different contexts.

From a practitioner's perspective, the key features of combined AO + MI therapy are that this method is essentially cost-free and does not require specialist delivery. Instead, implementation requires a simple change to clinical practice. As a matter of course, stroke rehabilitation practitioners regularly provide visual demonstrations of the actions they intend their patient to perform. As part of this process, it is less common for a practitioner to advise their patient on exactly *how* they should pay their attention and *what* features of the action they should focus on. Combined AO + MI instructions represent a straight forward way to formalise this important part of the rehabilitation programme. The recent review paper by Eaves et al. [33] suggested a number of critical considerations when using this technique in stroke rehabilitation, particularly from a research viewpoint. Here we make additional suggestions from a practical perspective on how to administer this technique.

Action observation is arguably more familiar to patients and demands less cognitive input compared to motor imagery. This argument is based on the idea that although AO can involve active attention (for example, when there is intention to imitate the observed action), it can also be passive in nature, operating predominantly on the basis of automatic processing mechanisms [81]. On the other hand, MI is largely a voluntary, purposeful and directed activity. It may therefore be more useful to focus on basic AO therapy in the early stages of rehabilitation. As the patient's ability and engagement in the task increases over time, it may be useful to gradually introduce the imagery component. To achieve this, it would be necessary to provide some basic training in how to engage in motor imagery. A number of protocols exist for structuring and priming the relevant imagery components, including the model for Physical, Environment, Task, Timing, Learning, Emotion, and Perspective (PETTLEP; [82]) imagery training. A technique that may then specifically help to bridge the gap between traditional AO therapy and traditional imagery training is Layered Stimulus Response Training (LSRT; [83]).

Based on bioinformational theory [84]–[85], LSRT is intended to help individuals more easily generate and control their imagery experience by breaking down the different elements of an image, before bringing them together again in layers that can become increasingly complex [31]. From this perspective, it would be useful to first introduce a visual demonstration of the action, which is later accompanied by specific instructions to imagine performing carefully selected action features, such as the velocity, the trajectory or the goal of the action, which are synchronised with the on-line display. Prior to this exercise, and through individual consultation, participants would identify the action features they find most vivid and accessible through their imagery. As their ability in this task increases, the same visual display would be used repeatedly, upon which it is possible to layer imagery content with additional action features that are increasingly more complex and vivid. While this can all be achieved as part of existing face-to-face provision, further opportunities arise through the incorporation of technology.

The use of simple smartphone apps aimed at health-related outcomes have increased both in health professionals and the general public [86]. Smartphones are invaluable for the education and management of health conditions [87] and, specifically within stroke patients, for providing: education, exercises, functional skills training, daily living activities, and assistive device tutorials [88]–[90]. Moreover, the National Clinical Guidelines for Stroke also encourage the use of smartphones within stroke care [91].

Apps can easily display interactive videos and pictures of actions that provide an opportunity to further implement and explore the effectiveness of combined AO + MI therapy in rehabilitative practice. This could either be in the presence of rehabilitation professionals, or independently while patients are alone, as long as the patient has the capacity to engage with this technology independently. App developers would therefore need to work closely with clinicians, researchers and stroke patients to refine the contents of the actions displayed and the tasks involved, so they are: 1) adaptable and cater for individual differences in ability for patients with various impairments following cerebral vascular accidents; 2) carefully regulated and integrated with accurate information and evidence based research; and 3) have the ability to collect data and provide feedback.

In addition to traditional rehabilitation methods, imagery instructions can also be employed when using more sophisticated methods, such as brain computer interface (BCI) technology. The aim of a BCI is to foster neuroplasticity through manipulation or self-regulation of neurophysiological activity facilitating motor recovery. This could be achieved through neurofeedback mechanisms that incorporate MI instructions. Using this approach, it is possible to control the movements of an on-screen cursor by recording the neural activity, for example, over the patient's motor cortex, while they imagine executing an action [92]. In a recent meta-analysis, Cervera et al. [93] showed evidence for BCI-induced functional and structural neuroplasticity at a subclinical level. Of the nine studies included in their review, which involved 235 stroke survivors in total, the motor improvements following BCI training exceeded the minimal clinically important difference, when quantified using the upper limb FMA tool. The summary effect size was also reported as ranging from medium to large.

A successful BCI setup depends on the user's ability to voluntarily control their own neural activity. Neuper et al. [59] was the first to investigate if the input signals required for effective BCI can be enhanced by combined AO + MI instructions. Using EEG methods, those authors showed AO + MI instructions produced significantly greater modulations of sensorimotor rhythms. This was when participants imagined performing a reach-grasp action while also observing visual feedback representing the same action, which was controlled using their own BCI input. This was compared to when participants purely imagined this task. This subtle but important change in protocol was therefore recommended as a more advantageous method for successful BCI. Given the recommendations in Cervera at al.'s [93] meta-analysis for future BCI research to engage with knowledge and understanding in the field of MI research, we would further recommend extending this to the literature investigating combined AO + MI methods. In doing so, it will be important to acknowledge the potential limits of these methods, which relate to a patient's capacity to engage with the technology, with respect to their level of cognitive and imagery ability.

## 4. Conclusion

There is now convincing evidence that combined AO + MI instructions elicit increased activity in motor regions of the brain, compared to either AO or MI independently. On these grounds, combined AO + MI therapy, in conjunction with physical practice, is recommended as a potentially more effective tool for practitioners in rehabilitation settings. While preliminary research indicates combined AO + MI therapy can be administered to stroke patients to directly impact neuroplasticity and motor outcomes [18], high quality research is now required to further validate this result. For practitioners, it is free and relatively easy to incorporate into their existing practice. It also represents an opportunity to prescribe useful home based training, which would be in addition to their one-to-one therapy sessions. Future research must now establish if this approach can help to improve motor recovery rates and facilitate independent rehabilitation. It is also important that future research establishes the best methods of delivery for combined AO + MI therapy, and how this should be tailored to the participant's ability and needs, while also exploring how this approach can be integrated with new technology that is both affordable and widely available.
